# Does investment in the health sector promote or inhibit economic growth?

**DOI:** 10.1186/1744-8603-9-43

**Published:** 2013-09-23

**Authors:** Aaron Reeves, Sanjay Basu, Martin McKee, Christopher Meissner, David Stuckler

**Affiliations:** 1 Department of Sociology, University of Oxford, Manor Road Building, Manor Road, Oxford OX1 3UQ, England; 2 Stanford Prevention Research Center, Department of Medicine, Stanford University, Palo Alto, CA, USA; 3 London School of Hygiene & Tropical Medicine, Department of Public Health and Policy, 15-17 Tavistock Place, London WC1H 9SH, UK; 4 Department of Economics, University of California Davis, Davis, USA

**Keywords:** Health spending, Government spending, Economic growth

## Abstract

**Background:**

Is existing provision of health services in Europe affordable during the recession or could cuts damage economic growth? This debate centres on whether government spending has positive or negative effects on economic growth. In this study, we evaluate the economic effects of alternative types of government spending by estimating “fiscal multipliers” (the return on investment for each $1 dollar of government spending).

**Methods:**

Using cross-national fixed effects models covering 25 EU countries from 1995 to 2010, we quantified fiscal multipliers both before and during the recession that began in 2008.

**Results:**

We found that the multiplier for total government spending was 1.61 (95% CI: 1.37 to 1.86), but there was marked heterogeneity across types of spending. The fiscal multipliers ranged from −9.8 for defence (95% CI: -16.7 to −3.0) to 4.3 for health (95% CI: 2.5 to 6.1). These differences appear to be explained by varying degrees of absorption of government spending into the domestic economy. Defence was linked to significantly greater trade deficits (β = −7.58, p=0.017), whereas health and education had no effect on trade deficits (p_education_=0.62; p_health_= 0.33).

**Conclusion:**

Our findings indicate that government spending on health may have short-term effects that make recovery more likely.

## Background

The Great Recession that beset Europe and North America since 2007 has sparked widespread debate about alternative approaches to achieving economic recovery. Much of the discussion has centred on the question of whether government spending will promote [1-3] or inhibit economic growth [4,5]. Critics of government spending argue, first, that such spending has an immediate effect of increasing debt which, if associated with a loss of confidence by investors, will also increase the cost of servicing that debt as a consequence of increased interest rates [6,7]. Second, they argue that, by “crowding out” private markets and, by implication, their assumed greater efficiency, they will inhibit the growth necessary for recovery [8]. In contrast, advocates of greater spending during recessionary periods, which are characterized by unemployment and deficits, argue that the effects of short term increases in borrowing will be compensated for by higher growth resulting from a high marginal propensity to consume, generating positive cycles of consumption and employment growth [9]. Many of these arguments have been tested empirically. Contrary to what is claimed by the advocates of austerity, increased debt is not consistently associated with high interest rates nor lower economic growth rates [10-12]. Similarly, rather than crowding out private markets, government spending, at least on physical infrastructure, seems to increase the productivity of private capital [13,14]. However, other aspects of stimulus spending have been subject to less attention from researchers.

One aspect that has received less attention is the potential return on government investment in the health sector. The fiscal multiplier is an estimate of the effect of government spending on economic growth. A multiplier greater than 1 corresponds to a positive growth stimulus (returning more than $1 for each dollar invested), whereas a multiplier less than one reflects a net loss from spending. Prior studies have estimated the aggregate multipliers from overall government spending [15-19], most notably in a recent study by two senior economists at the International Monetary Fund that suggested that the multiplier was greater than one, higher than the previously assumed multiplier of 0.5-0.6 [20]. It is obvious that some sectors will have greater capacity to translate additional funds into new employment opportunities or increase incomes domestically and thus intuitive that such expenditure will achieve greater fiscal multipliers [16,21]. However, to our knowledge no study has yet to comparatively estimate the growth effects of differing types of government spending.

While this paper cannot hope to resolve the debate between those advocating greater austerity to reduce levels of debt and those who see government spending as a way of encouraging recovery (and in turn the debt), it is possible to examine what any government that wished to adopt the latter course should spend its money on to achieve the greatest return on investment. Health, education, and social welfare sectors are currently experiencing significant cuts in several European countries, either as a result of political decisions by national governments or, in the case of countries subject to bail outs, by the troika of the European Commission, European Central Bank, and International Monetary Fund [22]. There is growing evidence that these cuts are having an adverse effect on the health of populations [23]. The question we ask here is whether they are also damaging the prospects for economic growth.

## Methods

In the first step of the analysis, we used comparative cross-national data on sector-specific funding [24] among 25 EU countries from 1995 to 2010 to estimate empirically the magnitude and direction of fiscal multipliers in health, defence, education, and other key government spending sectors (see Table 1 for definitions of budget categories).

**Table 1:** Types of government spending

**Types of government spending**	
Health	Government outlays on health include expenditures on services provided to individual persons and services provided on a collective basis; Medical products, appliances, and equipment; Outpatient, hospital, and public health services
Education	Pre-primary, primary, secondary, post-secondary, non-tertiary, tertiary education Provision of education not definable by level; Subsidiary services to education
Culture	Recreational, sporting, cultural, broadcasting, publishing, religious and other community services; R&D recreation, culture, and religion
Housing and community	Housing and community development; Water supply and street lighting
General public services	Executive and legislative organs, financial and fiscal affairs, external affairs, Public debt transactions; General and public services; Foreign economic aid Police, fire, and prison services; Law courts; R&D public order and safety
Defence	Military and civil defence; foreign aid defence; R&D Defence
Environment	Waste and waste water management; Pollution abatement. Protection of biodiversity and landscape
Social protection	Sickness and disability, old age, survivors, family and children, unemployment, housing, and social exclusion
Economic affairs	General economic, commercial, and labour affairs; agriculture, forestry, fishing, hunting, fuel and energy, mining, manufacturing, construction, transport, communication and other industries.

Source: EuroStat 2013 edition.

To estimate fiscal multipliers empirically in non-recession periods, we estimated a weighted-average of country-specific slopes (‘within-country’ variation). Government spending may correlate with unobserved factors that also independently affect economic growth. To address potential confounding factors, some studies apply mathematical simulation models, relying on theoretical assumptions [25,26]. Vector autoregressive models have been applied to quarterly data for small numbers of countries, but for annual data with larger numbers of countries fixed effects models are more consistent. Case-studies of small economies have been applied to correct for potential confounding factors, but these micro studies lack generalisability to larger economies. Instead, to correct for these potential confounding factors, we correct for total government spending as well as between-country heterogeneity by using fixed effects models [27,28], covering the years 1995–2007 as follows:

(1) $$\mathrm{GD}P_{\mathrm{it}}=\alpha +\beta G_{\mathrm{ijt}}+\varphi \left(\left(\sum_{j}G_{\mathrm{ijt}}\right)-G_{\mathrm{ijt}}\right)+\mu_{i}+\epsilon_{\mathrm{it}}$$

Here *i* is country, *j* is type of government spending as described in Table 1, and *t* is year. GDP is Gross Domestic Product and G is government spending, both in purchasing-power-parity and constant international 2005 dollars. β is the estimate fiscal multiplier, where β =1 is no effect, β >1 is a multiplicative effect, and β<1 is a contractionary effect, and φ is the fiscal multiplier for the total of all other types of government spending. Standard errors were clustered by country to reflect non-independence of sampling.

In the second stage of the analysis, to test whether fiscal multipliers differ in recession and non-recessionary periods, we quantified fiscal multipliers from the recessionary period between 2008 and 2010, then compared these estimated fiscal multipliers with those from pre-recession models. We also use out-of sample prediction with observed economic outcomes between 2008 and 2010 and for non-EU economies of the U.S. and Japan. In subsequent analyses, to assess whether specific multipliers differed across recessionary and non-recessionary periods, we added an interaction term for the recessionary years. All analyses were performed using STATA v12.1.

## Results

### Estimating fiscal multipliers by type of government spending, pre-recession 1995–2007

**Figure 1:**
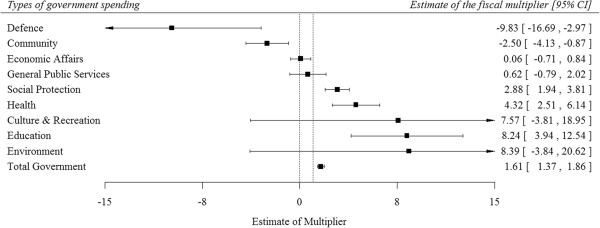
Pre-recession fiscal multipliers, 1995–2007.

The forest plot in Figure 1 displays the estimated fiscal multiplier across eight types of government spending, which range from −9.8 and 8.4 among various government spending sectors. Overall, we estimated the fiscal multiplier for total government spending as 1.61 (95% CI: 1.37 to 1.86), which is consistent with recent independent estimates of the aggregate multiplier [17].

The magnitude of fiscal multipliers differed significantly by type of government spending. As shown in Figure 1, we estimated a negative fiscal multiplier of defence spending (−9.8, 95% CI: -16.7 to −3.0), while the largest positive fiscal multipliers were in the sectors of health (4.32, 95% CI: 2.51 to 6.14) and education (8.24, 95% CI: 3.94 to 12.54).

**Figure 2:**
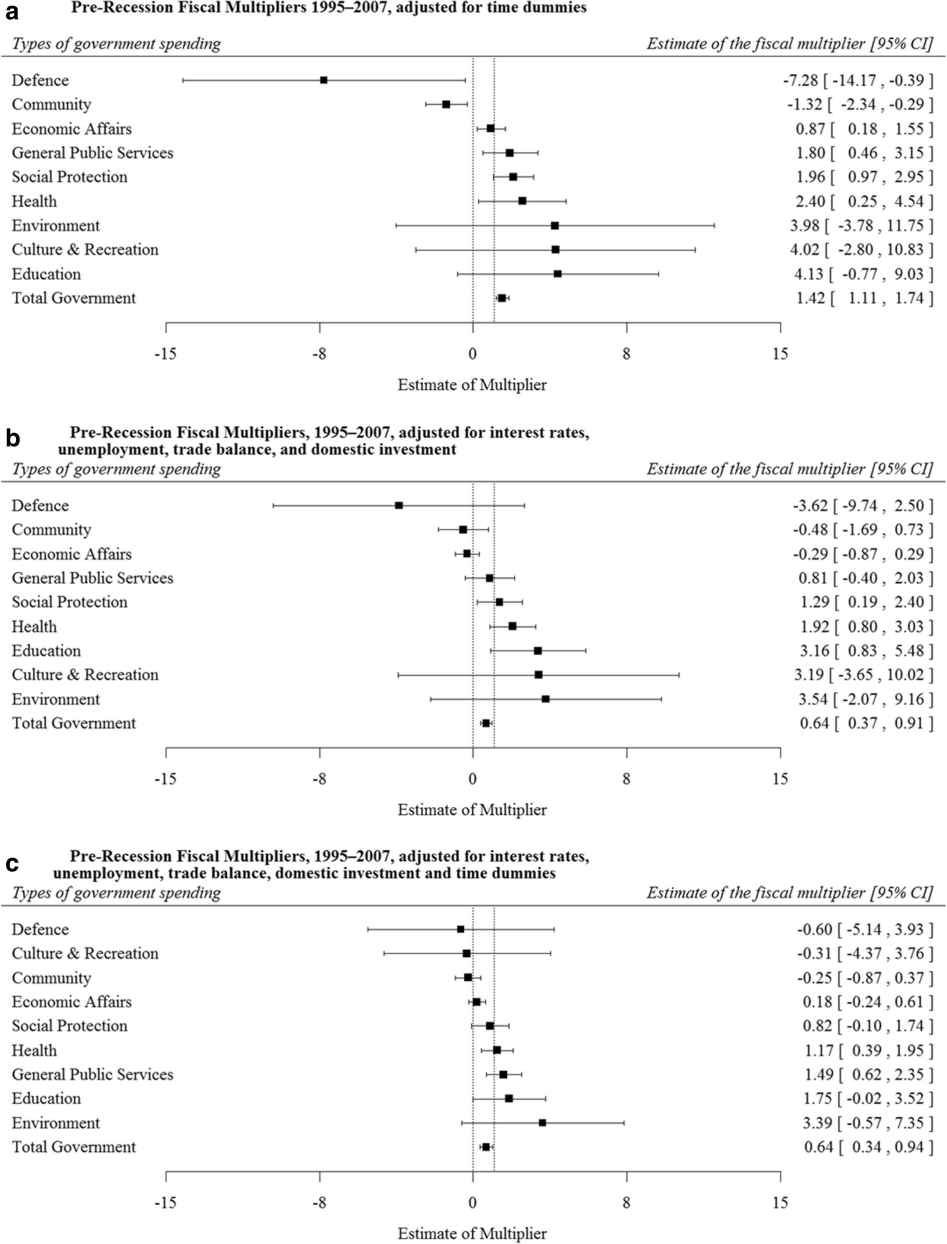
**Adjusted pre-recession fiscal multipliers 1995–2007, by type of government spending. a**. Pre-recession fiscal multipliers 1995–2007, adjusted for time dummies, 1995–2007. **b**. Pre-recession fiscal multipliers, 1995–2007, adjusted for interest rates, unemployment, trade balance, and domestic investment. **c**. Pre-recession fiscal multipliers, 1995–2007, adjusted for interest rates, unemployment, trade balance, domestic investment and time dummies.

To further evaluate potential mediating factors, we included a standard set of determinants of economic growth, including time dummies, interest rates, unemployment, trade balance, and domestic investment (Figure 2a). All factors had associations in the expected direction (e.g. higher unemployment and interest rates were negative, whereas greater savings, investment, and net exports were positive) (see Tables 2a-j). Consistent with a domestic multiplier mechanism, we found that when we adjusted for unemployment, the health and education multipliers were attenuated, but adjusting for trade balance had no effect. In contrast, when after adjusting for the trade balance, the defence spending multiplier was attenuated (β = − 3.62, Table 2c).

**Table 2:** Estimated fiscal multipliers, adjusted for unemployment rates, interest rates, trade balance (net exports), and domestic investment

**a. Fiscal multiplier for total government spending**					
	**Gross domestic product adjusted for inflation and purchasing-power parity**				
**Covariates**	**(1)**	**(2)**	**(3)**	**(4)**	**(5)**
Total government spending	1.61**	1.60**	1.38**	1.10**	0.64**
	(0.12)	(0.15)	(0.17)	(0.10)	(0.13)
Unemployment rate	—	−248.28**	−258.67**	−364.58**	−205.14
		(65.84)	(86.17)	(94.99)	(131.78)
Interest rate	—	—	−320.26**	−304.69**	−269.42**
			(94.24)	(76.57)	(54.26)
Net exports (PPP, Real, per capita)	—	—	—	0.61**	1.31**
				(0.16)	(0.19)
Total domestic investment (PPP, Real, per capita)	—	—	—	—	1.12**
					(0.26)
Country-years	296	213	213	213	192
Number of countries	25	19	19	19	19
**b. Fiscal multiplier for health spending**					
	**Gross domestic product adjusted for inflation and purchasing-power parity**				
**Covariates**	**(1)**	**(2)**	**(3)**	**(4)**	**(5)**
Total health spending	4.32**	3.21**	2.07	2.23*	1.92**
	(0.88)	(0.98)	(1.21)	(0.90)	(0.53)
Total government spending minus health spending	1.05**	1.26**	1.25**	0.88**	0.39*
	(0.33)	(0.37)	(0.39)	(0.27)	(0.15)
Unemployment rate	—	−205.61**	−239.03*	−336.54**	−173.88
		(57.89)	(87.57)	(94.16)	(123.10)
Interest rate	—	—	−286.44**	−248.57**	−203.53**
			(83.05)	(58.98)	(47.45)
Net exports (PPP, Real, per capita)	—	—	—	0.64**	1.24**
				(0.17)	(0.17)
Total domestic investment (PPP, Real, per capita)	—	—	—	—	1.06**
					(0.25)
Country-years	296	213	213	213	192
Number of countries	25	19	19	19	19
**c. Fiscal multiplier for defense spending**					
	**Gross domestic product adjusted for inflation and purchasing-power parity**				
**Covariates**	**(1)**	**(2)**	**(3)**	**(4)**	**(5)**
Total defense spending	−9.83**	−13.17**	−12.92**	−8.51	−3.62
	(3.32)	(3.67)	(4.33)	(4.77)	(2.91)
Total government spending minus defense spending	1.66**	1.62**	1.42**	1.20**	0.71**
	(0.08)	(0.09)	(0.10)	(0.11)	(0.14)
Unemployment rate	—	−286.58**	−301.73**	−368.95**	−221.27
		(66.15)	(90.73)	(91.27)	(124.46)
Interest rate	—	—	−253.31*	−263.02**	−251.73**
			(96.90)	(84.62)	(55.96)
Net exports (PPP, Real, per capita)	—	—	—	0.47*	1.24**
				(0.20)	(0.18)
Total domestic investment (PPP, Real, per capita)	—	—	—	—	1.08**
					(0.25)
Country-years	296	213	213	213	192
Number of countries	25	19	19	19	19
**d. Fiscal multiplier for education spending**					
	**Gross domestic product adjusted for inflation and purchasing-power parity**				
**Covariates**	**(1)**	**(2)**	**(3)**	**(4)**	**(5)**
Total education spending	8.24**	6.53**	4.98	5.06**	3.16*
	(2.09)	(2.10)	(2.50)	(1.75)	(1.11)
Total government spending minus education spending	0.66*	0.88**	0.92**	0.59**	0.33
	(0.24)	(0.26)	(0.27)	(0.20)	(0.24)
Unemployment rate	—	−188.09**	−227.19**	−333.20**	−168.63
		(58.30)	(76.03)	(84.09)	(117.61)
Interest rate	—	—	−211.45	−184.21	−186.19**
			(127.38)	(97.00)	(61.05)
Net exports (PPP, Real, per capita)	—	—	—	0.63**	1.26**
				(0.15)	(0.17)
Total domestic investment (PPP, Real, per capita)	—	—	—	—	1.11**
					(0.22)
Country-years	296	213	213	213	192
Number of countries	25	19	19	19	19
**e. Fiscal multiplier for general public services spending**					
	**Gross domestic product adjusted for inflation and purchasing-power parity**				
**Covariates**	**(1)**	**(2)**	**(3)**	**(4)**	**(5)**
Total general public services spending	0.62	1.37	2.44*	2.05*	0.81
	(0.68)	(0.71)	(0.89)	(0.88)	(0.58)
Total government spending minus general public services spending	1.75**	1.63**	1.21**	0.96**	0.62**
	(0.09)	(0.12)	(0.14)	(0.14)	(0.11)
Unemployment rate	—	−242.48**	−279.25**	−381.55**	−207.43
		(67.58)	(88.70)	(100.42)	(132.83)
Interest rate	—	—	−396.26**	−372.92**	−284.09**
			(97.04)	(84.44)	(59.63)
Net exports (PPP, Real, per capita)	—	—	—	0.60**	1.30**
				(0.16)	(0.19)
Total domestic investment (PPP, Real, per capita)	—	—	—	—	1.12**
					(0.27)
Country-years	296	213	213	213	192
Number of countries	25	19	19	19	19
**f. Fiscal multiplier for culture&recreation spending**					
	**Gross domestic product adjusted for inflation and purchasing-power parity**				
**Covariates**	**(1)**	**(2)**	**(3)**	**(4)**	**(5)**
Total culture & recreation spending	7.57	6.25	4.52	0.83	3.19
	(5.51)	(4.70)	(3.99)	(2.96)	(3.25)
Total government spending minus culture & recreation spending	1.34**	1.38**	1.24**	1.11**	0.57**
	(0.32)	(0.30)	(0.26)	(0.19)	(0.18)
Unemployment rate	—	−243.28**	−259.08**	−365.32**	−194.27
		(65.52)	(88.99)	(94.63)	(129.50)
Interest rate	—	—	−306.23**	−305.79**	−258.23**
			(98.71)	(77.59)	(54.23)
Net exports (PPP, Real, per capita)	—	—	—	0.61**	1.31**
				(0.16)	(0.18)
Total domestic investment (PPP, Real, per capita)	—	—	—	—	1.14**
					(0.26)
Country-years	296	213	213	213	192
Number of countries	25	19	19	19	19
**g. Fiscal multiplier for community spending**					
	**Gross domestic product adjusted for inflation and purchasing-power parity**				
**Covariates**	**(1)**	**(2)**	**(3)**	**(4)**	**(5)**
Total community spending	−2.50**	−2.32**	−1.57**	−0.91	−0.48
	(0.79)	(0.63)	(0.52)	(0.63)	(0.58)
Total government spending minus community spending	1.68**	1.67**	1.47**	1.19**	0.73**
	(0.10)	(0.13)	(0.15)	(0.09)	(0.11)
Unemployment rate	—	−231.99**	−255.56**	−351.38**	−198.19
		(58.31)	(85.00)	(90.87)	(129.65)
Interest rate	—	—	−254.50**	−260.82**	−248.63**
			(80.86)	(69.72)	(49.31)
Net exports (PPP, Real, per capita)	—	—	—	0.55**	1.22**
				(0.14)	(0.19)
Total domestic investment (PPP, Real, per capita)	—	—	—	—	1.03**
					(0.28)
Country-years	296	213	213	213	192
Number of countries	25	19	19	19	19
**h. Fiscal multiplier for environment spending**					
	**Gross domestic product adjusted for inflation and purchasing-power parity**				
**Covariates**	**(1)**	**(2)**	**(3)**	**(4)**	**(5)**
Total environment spending	8.39	1.03	−0.46	3.02	3.54
	(5.92)	(4.73)	(4.01)	(4.02)	(2.67)
Total government spending minus environment spending	1.50**	1.60**	1.40**	1.07**	0.59**
	(0.17)	(0.16)	(0.18)	(0.14)	(0.14)
Unemployment rate	—	−251.28**	−269.12**	−356.05**	−193.58
		(67.63)	(86.71)	(96.24)	(128.60)
Interest rate	—	—	−322.65**	−301.83**	−261.57**
			(94.54)	(78.69)	(56.92)
Net exports (PPP, Real, per capita)	—	—	—	0.62**	1.35**
				(0.16)	(0.18)
Total domestic investment (PPP, Real, per capita)	—	—	—	—	1.14**
					(0.26)
Country-years	296	213	213	213	192
Number of countries	25	19	19	19	19
**i. Fiscal multiplier for economic affairs spending**					
	**Gross domestic product adjusted for inflation and purchasing-power parity**				
**Covariates**	**(1)**	**(2)**	**(3)**	**(4)**	**(5)**
Total economic affairs spending	0.06	−0.06	0.03	−0.55	−0.29
	(0.38)	(0.45)	(0.40)	(0.35)	(0.28)
Total government spending minus economic affairs spending	1.83**	1.80**	1.58**	1.32**	0.84**
	(0.14)	(0.17)	(0.20)	(0.14)	(0.19)
Unemployment rate	—	−248.83**	−271.11**	−388.53**	−233.36
		(62.40)	(75.94)	(84.40)	(125.08)
Interest rate	—	—	−265.45**	−237.34**	−238.72**
			(91.29)	(74.14)	(50.65)
Net exports (PPP, Real, per capita)	—	—	—	0.66**	1.27**
				(0.13)	(0.18)
Total domestic investment (PPP, Real, per capita)	—	—	—	—	0.97**
					(0.26)
Country-years	296	213	213	213	192
Number of countries	25	19	19	19	19
**j. Fiscal multiplier for social protection spending**					
	**Gross domestic product adjusted for inflation and purchasing-power parity**				
**Covariates**	**(1)**	**(2)**	**(3)**	**(4)**	**(5)**
Total social protection spending	2.88**	3.25**	2.72**	2.21**	1.29*
	(0.45)	(0.49)	(0.64)	(0.49)	(0.53)
Total government spending minus social protection spending	0.83**	0.52	0.57	0.47*	0.36
	(0.27)	(0.26)	(0.31)	(0.20)	(0.20)
Unemployment rate	—	−289.73**	−323.90**	−410.46**	−254.49
		(61.01)	(85.98)	(94.19)	(142.36)
Interest rate	—	—	−211.82	−217.75*	−229.26**
			(101.48)	(80.13)	(59.17)
Net exports (PPP, Real, per capita)	—	—	—	0.57**	1.28**
				(0.17)	(0.21)
Total domestic investment (PPP, Real, per capita)	—	—	—	—	0.98**
					(0.31)
Country-years	296	213	213	213	192
Number of countries	25	19	19	19	19

*Notes*: Robust standard errors in parentheses clustered by country to reflect non-independence of sampling.

** p<0.01, * p<0.05.

We found evidence that the size of the fiscal multiplier was partly mediated by the degree of domestic absorption of government spending. Tradable components of government spending, like defence spending, were linked to significantly more negative trade balance (β = −7.58, p=0.017) (see Additional file 1: Web Appendix 1). However, non-tradable areas of the budget, including health and education had no significant association with the trade balance (p_education_=0.62; p_health_= 0.33) (see Additional file 1: Web Appendix 1).

### Observed fiscal multiplier for total government spending, 2008–2010

**Figure 3:**
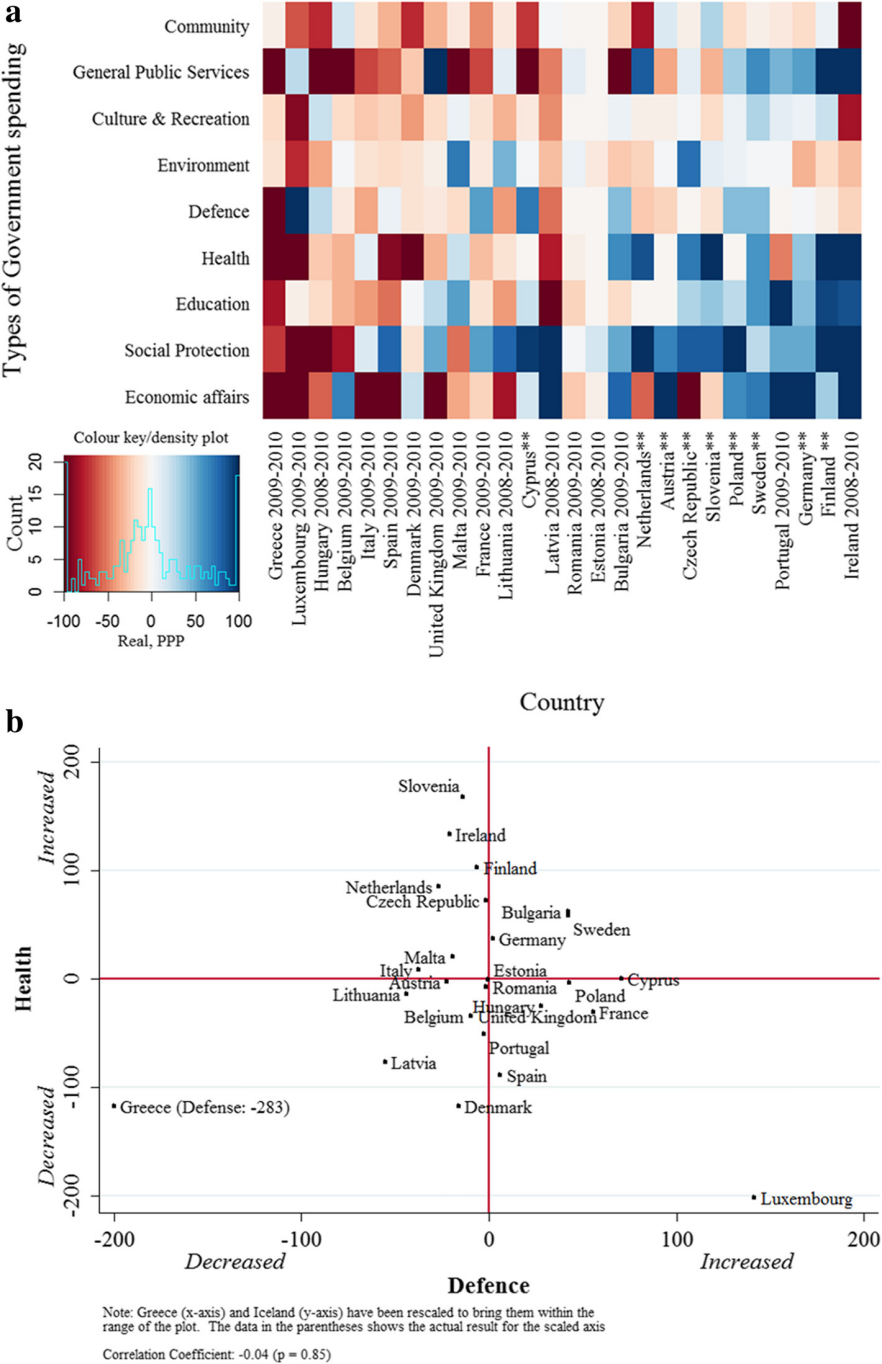
**Budgetary patterns and effects. Panel a**. Heat map of budget changes in Europe. **Panel b**. Association of health with defence spending.

As a final test, we performed two out-of-sample predictions using our estimates from the 1995–2007 period. First, we quantified the fiscal multiplier for total government spending during the current recessionary period by identifying the period of significant changes in government spending. Panel a of Figure 3 presents a heat map showing patterns of government spending change in the EU (blue = increased, red = decreased spending). Europe’s governments have differed considerably in their patterns of expenditure, both overall and across sectors. Greece, Luxembourg, and Hungary have implemented large reductions, whereas Germany, Finland, Poland, and Sweden implemented significant spending increases. In general, community and general public services (e.g. civil service) have seen the largest budget shifts in both positive and negative directions. Social protection spending has tended to increase during the recession, while defence spending has tended to decline slightly.

**Figure 4:**
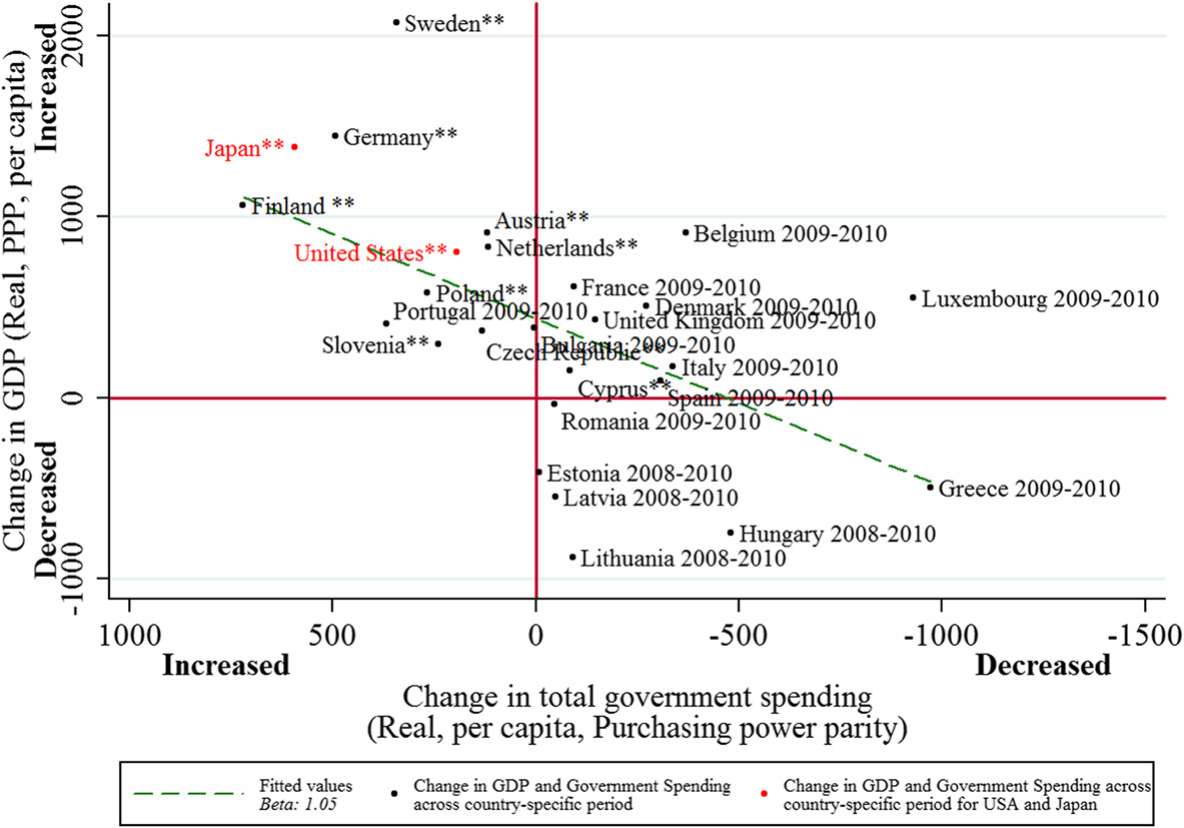
Recessionary fiscal multiplier, 2008–2010.

Figure 4 presents the association of changes in government spending and in Gross Domestic Product (β = 1.05, 95% CI: 0.49 to 1.61, p<0.01) during the current recession. As shown in the figure, those countries which have instituted greater increases in government spending have had larger rises in per capita GDP.

Second, to further test the generalizability of our findings, we performed an out-of-sample prediction using GDP data on the US and Japan. As shown in Figure 4, during the recessionary years of 2008–2010, both US and Japan’s economic performance were predicted by changes in total government spending.

### Comparing recession and pre-recession multipliers

To test whether fiscal multipliers differed across recessionary and non-recessionary periods, we extended our disaggregated analysis of fiscal multipliers of 1995–2007 to include 2008–2010. As shown in Table 3, there was a significant interaction of the recessionary period with the estimated magnitude of fiscal multipliers. Apart from defence and public service spending, the fiscal multipliers were slightly attenuated during the recessionary period. The multiplier for total government spending, for example, was reduced by 0.15 (95% CI: 0.08 to 0.21). Health and education dropped, respectively, by 1.31 (95%: 0.94 to 1.68) and 1.44 (95% CI: 0.80 to 2.08). Social protection spending was reduced to a lesser degree, by 0.33 (95%: 0.11 to 0.56). However, in the statistical models, the health, education, and social protection categories of spending all exhibited statistically significant and positive growth associations above 1 during the recessionary period.

**Table 3:** Recessionary (1995–2010) and pre-recession fiscal multipliers (1995–2007), by type of government spending

	**Increase in GDP (PPP, per capita, real)**		
**Covariates**	**Estimated multiplier 1995-2010**	**Recession interaction**	**Estimated recession multiplier**
	(1)	(2)	(3)
Total Government Spending	1.28	−0.15	1.13
	(0.78 to 1.77)	(−0.084 to −0.21)	(0.66 to 1.61)
Defence	−5.69	−1.71	−7.40
	(−15.77 to 4.39)	(−6.16 to 2.74)	(−18.12 to 3.32)
Community	−2.29	−5.99	−8.29
	(−4.84 to 0.25)	(−1.34 to −10.64)	(−13.45 to −3.12)
Economic Affairs	0.45	−1.17	−0.72
	(−0.35 to 1.24)	(−1.99 to −0.35)	(−1.09 to −0.35)
General Public Services	1.57	−0.45	1.12
	(−0.26 to 3.40)	(−1.17 to 0.28)	(−0.75 to 3.00)
Social Protection	3.04	−0.33	2.71
	(2.05 to 4.03)	(−0.56 to −0.11)	(1.81 to 3.60)
Health	4.92	−1.31	3.61
	(2.92 to 6.93)	(−1.68 to −0.94)	(1.60 to 5.62)
Culture & Recreation	14.12	−2.38	11.83
	(2.16 to 26.26)	(−0.67 to −4.10)	(−0.92 to 24.58)
Education	9.37	−1.44	7.92
	(4.40 to 14.34)	(−2.08 to −0.80)	(3.19 to 12.66)
Environment	9.49	−6.30	3.18
	(−5.03 to 24.00)	(−8.93 to −3.68)	(−10.60 to 16.97)

*Notes*: Numbers in parentheses are 95% confidence intervals. GDP and all forms of government spending are adjusted for inflation and purchasing-power parity. Robust standard errors in parentheses clustered by country to reflect non-independence of sampling Columns 1–2 report coefficients from equation 2. Column 3 reports the linear combination of columns 1 and 2. All models control for between-country variation.

### Sensitivity analysis

We tested the robustness of multiplier estimates to several alternative model specifications. First, we incorporated a set of time dummies to correct for cross-EU economic interactions, finding that none of the results was qualitatively changed (see Figure 2b). Second, we included both time dummies and the aforementioned set of controls and found that direction of the coefficients remained the same and the health multiplier remained above 1 in the pre-recessionary period (see Figure 2c). Third, we used alternative data sources of government statistics, including the OECD and World Bank. None of the spending multipliers qualitatively changed.

To test whether budgetary changes in the recessionary period were confounded by the depth of recession (peak-to-trough change in GDP), we assessed the correlation of depth of recession and the depth of subsequent government spending changes (r = 0.12, p = 0.59). None of the results was changed when controlling for the cumulative magnitude of recession. Overall, we observed no correlation among changes in government spending instituted in different sectors (e.g., changes in health and defence spending are not correlated, r = 0.02, p =0.90, panel b of Figure 3), indicating large, exogenous variation in budgetary choices in response to recession.

## Discussion

Government spending changes during recession serves as a large “quasi-natural experiment”, in which countries experiencing relatively similar recessions undertook alternative budgetary paths. From these differences, we can deduce the relationship between spending and economic outcomes. Our findings suggest a total pro-growth effect of overall government spending, with significant positive fiscal multipliers in the social protection, health and education sectors [29]. These estimates were based on cross-national models addressing potential economic and political confounding factors. By empirically evaluating fiscal multipliers across different sectors, we observed that changes in government spending corresponded to similar directional changes in economic growth during non-recessionary and recessionary periods. Multipliers estimated from non-recession periods can account for current changes in economic growth associated with spending instituted in 2008–2010.

Our study has several limitations. First, the statistical analysis covers only the first three years of economic recession, given the limited availability of more recent data. While it has been argued that short-term multipliers may be fail to account for longer-term effects [30], we found that estimated fiscal multipliers from 1995–2007 predicted directional changes in subsequent short-term periods. However, it is possible that longer-term effects differ from those observed currently, remaining an important topic for future research. Second, there was marked heterogeneity in the magnitude of fiscal multipliers. This heterogeneity exists both within and between countries. Within countries, our results identified that domestic absorption of spending is a significant factor mediating the relationship between government spending and economic growth. When we adjusted for the trade balance, the multipliers for components of government spending which are tradable, such as defence, were attenuated, whereas semi-tradable components, such as health and education, were not significantly altered. Across countries, the magnitude of the fiscal multiplier is also likely to depend on several country-specific factors and market characteristics, including the level of economic development, exchange rate regime, openness to trade, and public debt dynamics [31,32]. Third, the data on government spending could not differentiate the growth effects of government spending that acts as a stimulus from those effects of spending which replaces spending during recessions. Although it has been suggested that recessionary periods exhibit higher multiplier effects, as consumer demand is relatively low, we found contrary evidence that fiscal multipliers were slightly attenuated during periods of recessions compared with periods of economic growth. This may reflect a tendency of ‘replacement’ spending, such as when patients turn from private health care to public health care consumption during recessions, to have smaller pro-growth effects than ‘stimulus’ spending, which creates new jobs and income. This interpretation is corroborated by the observation that health spending was more greatly attenuated than total social protection, as the latter is largely driven by replacement spending both in recessionary and non-recessionary periods. Another possibility is that when multiple countries simultaneously introduce budget reductions, as in the current recession, the potential multiplier effects of government spending are dampened. Further disaggregating categories of government spending, identifying time lags, and evaluating the role of effect modifiers associated with various forms of government spending would be a next logical step for research to identify critical policy strategies during and after the ongoing economic recession.

Overall, our findings are consistent with recent estimates of fiscal multipliers for total government spending [28], but extends these estimates in a novel way to evaluate alternative budgetary sectors, compare periods of non-recession and recession, identify the role of domestic absorption, and include both Europe and the United States. It also lends support for theories that there are both ‘productive’ and ‘unproductive’ components of government spending [33]. However, in contrast with previous case-study estimates of the fiscal multipliers associated with defence spending in the United States [3,18,19], we estimated negative fiscal multipliers for military spending in Europe. This observation may reflect the presence of a large defense manufacturing sector in the United States so that most expenditure is absorbed domestically while, in most European countries, equipment is imported. It is also plausible that military spending will vary according to where the funds are being spent, so the multiplier effect may be greater in countries with a large domestic defence industry.

These findings have important implications for policy. First, these results, taken together with other studies, corroborates existing evidence that historical prescriptions for austerity from international financial institutions have tended to exacerbate economic crises [29,34,35]. Second, there is a widespread consensus that investment in education and in health contribute to economic growth in the long term, by creating a healthier, better educated, and therefore more productive labour force [36]. However, that argument finds little favour among those who view short term reductions in expenditure as a necessary condition for the recovery that will permit such investment in the future. Our findings suggest that, in addition to their long-term benefits, such investments may actually have short-term, positive growth effects that make that recovery more likely.

## Competing interests

We declare that we have no conflicts of interests.

## Authors’ contributions

AR DS analyzed the data and wrote the draft; SB MM CM assisted with the analysis and helped finalise the paper. All authors read and approved the final manuscript.

## Supplementary Material

Additional file 1 **Web Appendix 1.** Associations between trade balance and sector-specific government spending between 1995–2007, 26 EU countries. Click here for file
