# Supplementary material for: Does investment in the health sector promote or inhibit economic growth?
Source: Global Health. 2013 Sep 23;9:43. doi: 10.1186/1744-8603-9-43 (PMC3849102; doi:10.1186/1744-8603-9-43)
Supplement: Additional file 1 — Web Appendix 1. Associations between trade balance and sector-specific government spending between 1995–2007, 26 EU countries. [file 1744-8603-9-43-S1.doc]

Web Appendix 1. Associations between trade balance and sector-specific government spending between 1995-2007, 26 EU countries.

| Types of government spending | Trade balance |
| --- | --- |
| Total Government Spending | 0.36 (0.26) |
| Defence | -7.58^*^ (2.96) |
| Community | -1.78 (1.32) |
| Economic Affairs | 0.13 (0.11) |
| General Public Services | 0.75 (0.59) |
| Social Protection | 1.38 (0.96) |
| Health | -0.87 (0.88) |
| Culture & Recreation | 7.62 (4.71) |
| Education | 0.77 (1.53) |
| Environment | -0.56 (3.65) |
| Country-years | 382 |
| Number of countries | 25 |

*Notes:* Standard errors are adjusted for repeated observations within countries. All models adjust for unobserved between country variation. A negative trade balance indicates that imports exceed exports.
